# Are behavioral responses to eyespots in sticklebacks influenced by the visual environment? An experimental examination

**DOI:** 10.1002/ece3.9089

**Published:** 2022-07-05

**Authors:** Evelina Juntorp, Madicken Åkerman, John L. Fitzpatrick

**Affiliations:** ^1^ Department of Zoology: Ethology Stockholm University Stockholm Sweden

**Keywords:** anti‐predator coloration, eyespots, natural selection, predation, startle displays

## Abstract

Eyespots are taxonomically widespread color patterns consisting of large concentric rings that are commonly assumed to protect prey by influencing the behaviors of predators. Although there is ample experimental evidence supporting an anti‐predator function of eyespots in terrestrial animals, whether eyespots have a similar deterring function in aquatic animals remains unclear. Furthermore, studies in terrestrial systems suggest that the protective function of eyespots depends on ambient light conditions where predators encounter them, but this effect has never been tested in aquatic environments. Here, we examine how eyespots influence behavioral responses in an aquatic environment under different visual environments, using laboratory‐reared three‐spined sticklebacks (*Gasterosteus aculeatus*) as model predators. Specifically, we experimentally examined behavioral responses of sticklebacks toward artificial prey patterns (control vs. eyespots) under two different light environment treatments (low vs. high). We found that eyespots did not postpone attacks from sticklebacks. However, sticklebacks approaching eyespots stopped more frequently than sticklebacks approaching prey items with a control pattern. Sticklebacks were (marginally) slower to attack prey in the low‐light treatment, but the light level did not influence stickleback behavioral responses toward eyespots. We conclude that eyespots can modulate some behaviors of an aquatic predator, albeit with a different functional role from that previously demonstrated in terrestrial species.

## Graphical abstract

Sticklebacks perform more stop behaviours as they approach prey with eyespot patterns. This suggests that eyespots may play a functional role in deterring predators in aquatic environments.

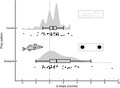

## 1 INTRODUCTION

Predation is a powerful selective force that profoundly impacts animal phenotypes (Cott, 1940; Pembury Smith & Ruxton, 2020; Stevens & Merilaita, 2011; Stevens & Ruxton, 2019). In particular, the wide range of remarkable body coloration and patterns observed in animals are often attributed to a functional role in reducing the likelihood of predation (Barnard, 1983; Cuthill et al., 2017; Poulton, 1890). One type of body pattern that has attracted substantial and sustained research attention is eyespots (Cott, 1940; Poulton, 1890). Eyespots are conspicuous circular markings that diverge from the surrounding body pattern, commonly with concentric rings of contrasting colors that are often assumed to resemble a vertebrate eye and have an anti‐predator function (Blest, 1957; Stevens, 2005). Eyespots may reduce predation risk by intimidating or startling potential predators either by resembling the eye of a potential predator (the intimidation hypothesis, Blest, 1957; De Bona et al., 2015; Merilaita et al., 2011; Vallin et al., 2005, 2007) or simply by being conspicuous (Stevens, 2005, 2007; Stevens et al., 2009), signaling to predators that they have been detected by potential prey (the detection hypothesis, Caro, 1995; Radford et al., 2020), or by diverting predator strikes to less vital or posterior body parts (the deflection hypothesis, Hill & Vaca, 2004; Prudic et al., 2015; Vallin et al., 2011). Yet, despite being taxonomically widespread (Poulton, 1890), the vast majority of work on eyespots has focused on butterflies with avian predators (Stevens, 2005), leading to calls to investigate the potential anti‐predator function of eyespots in a wider range of animals (e.g., Hemingson et al., 2021; Kjernsmo & Merilaita, 2013; Kelley et al., 2013).

Since the pioneering work by Blest (1957), the impact of eyespots on predator behaviors has been demonstrated repeatedly by comparing predatory behavior and predation success on prey with or without eyespots (e.g., Kodandaramaiah et al., 2009; Olofsson et al., 2013; Prudic et al., 2015; Vallin et al., 2006, 2005, 2011). However, how eyespots influence predator behaviors is often dependent on the visual environment where predators would encounter them (Lyytinen et al., 2003; Stevens et al., 2008). For example, wing spots on artificial moths reduce predation risk when prey are conspicuous to the background, but not on camouflaged prey (Stevens et al., 2008). Similarly, artificial caterpillars with eyespots are attacked less frequently only if they are counter shaded (Hossie & Sherratt, 2012). Furthermore, the efficacy of eyespots in deterring predation can depend on an interactive effect with the surrounding habitat (Hossie et al., 2015), suggesting that the anti‐predator function of eyespots is highly context dependent (Lyytinen et al., 2003). In butterflies, for example, the impact of eyespots on predator behaviors is hypothesized to be critical during times of, or in habitats with, poor light conditions, when low temperatures hinder the ectothermic butterflies from escape attempts (Olofsson et al., 2010). Indeed, Olofsson et al. (2010) showed that eyespots of the woodland brown butterfly (*Lopinga achine*) were only effective in deflecting attacks from blue tits (*Cyanistes caeruleus*) in a laboratory environment under low‐light conditions. Thus, light conditions can be crucial for the anti‐predator function of eyespots and emphasize the importance of studying behavioral responses of predators to eyespots under different visual contexts.

Eyespots are found in many fish species and, as is the case in terrestrial species, are usually assumed to have an anti‐predator function (Cott, 1940; Hemingson et al., 2021; Kjernsmo & Merilaita, 2013; Kelley et al., 2013; Meadows, 1993; Winemiller, 1990). However, only a handful of studies have experimentally investigated the anti‐predator function of eyespots in aquatic animals. Experimental work has shown that small eyespots are effective in diverting the strikes of attacking three‐spined stickleback *Gasterosteus aculeatus*, which is consistent with the deflection hypothesis (Kjernsmo et al., 2016, 2019). Sticklebacks are also more hesitant to attack an eye‐like marking, consistent with the intimidation hypothesis (Kjernsmo & Merilaita, 2017), albeit not toward markings resembling an eyespot with the typical circular shape (Kjernsmo & Merilaita, 2013). Therefore, whether the “typical” eyespots of some aquatic prey deter predators in aquatic environments remains unclear.

Moreover, although the light environment varies by day, water depth, and season in aquatic environments (Lythgoe, 1988), it is not clear whether light levels influence how predators respond to eyespots. Predation risk is typically linked with light levels in aquatic environments, as piscivores are often more active during conditions of low‐light levels (e.g., at dawn and dusk, Brown & Magnavacca, 2003; Cerri, 1983; Helfman, 1979; Hobson, 1979; Major, 1977). Thus, fish may be more cautious when approaching eyespots under low‐light conditions, particularly in cases where eyespots function to provide cues of a potential predator. Alternatively, the detectability of eyespots may be reduced under low‐light conditions, which could reduce their impact on predator behaviors. For example, under low‐light intensities, prey detection time increases (e.g., Richmond et al., 2004) and reaction distances to predators are reduced (e.g., Mazur & Beauchamp, 2003), indicating that low‐light levels can impact both predator and prey behaviors. Consequently, testing the role of eyespots in an aquatic environment offers an ecological relevant opportunity to examine how light conditions might alter behavioral responses toward eyespots.

In this study, we experimentally examined behavioral reactions of three‐spined stickleback *Gasterosteus aculeatus* toward artificial prey stimuli with different patterns (control vs. eyespots) under two light environment treatments (low vs. high light). Stickleback were chosen as a model as they are visual predators (Ibrahim & Huntingford, 1989; Litvak & Leggett, 1992), are relatively easy to maintain in the lab, and respond to eyespots in experimental settings (Kjernsmo & Merilaita, 2013, 2017; Kjernsmo et al., 2016, 2019). Stickleback are commonly predated by larger piscivorous fish (e.g., perch, *Perca fluviatilis*), and eyespots (when larger than the stickleback's own eye) are commonly hypothesized to mimic the eyes of such piscivores (Kjernsmo & Merilaita, 2017). Consistent with the proposed anti‐predator function of eyespots, we predicted that sticklebacks would take longer to attack prey and approach prey more cautiously when presented with the eyespot compared to the control prey pattern. We also hypothesized that eyespots will differentially impact stickleback attacks under different light conditions.

## 2 MATERIAL AND METHODS

### 2.1 Experiment with animals and housing conditions

We conducted a laboratory experiment with first‐generation offspring of wild‐caught three‐spined sticklebacks (*Gasterosteus aculeatus*) captured in the Baltic Sea at Öresund, Sweden, in December 2018. Fertilized eggs were obtained from nests and hatched in isolation from adults in May 2019 at Stockholm University, Sweden. After hatching, juveniles were held in a continuously filtered and aerated artificial brackish (salinity: 0.5%) 1200‐L aquarium with a water temperature of 20°C, gravel on the bottom, and ceramic pots and plastic tubes for hiding space. Fish were fed daily, initially with live *Artemia salina* nauplii and later with frozen bloodworms and mysid shrimp. By using laboratory‐reared offspring (rather than wild‐caught fish), we generated experimental fish free from the tapeworm *Schistocephalus solidus*, a parasite that alters stickleback feeding activity and predator avoidance behaviors (Giles, 1987; Godin & Sproul, 1988). Thus, we could ensure that behavioral responses in our experiments were not due to confounding effects caused by the parasite. As our experiment was dependent on foraging behavior, fish were transitioned from a long photoperiod length (L:16, D:8) and at approximately 2 months of age, held at a short photoperiod length (L:8, D:16) to inhibit sexual maturation (Shao et al., 2013) that otherwise could have influenced foraging behavior (Worgan & FitzGerald, 1981). In October 2019, adult fish (size range: 37–45 mm) were moved to Tovetorp Zoological Research Station, a part of Stockholm University, where the experiment was performed between April and June 2020. The study was performed with permission from Stockholm ethical committee (decision numbers: 13362–2019).

### 2.2 Experimental set‐up

**FIGURE 1:**
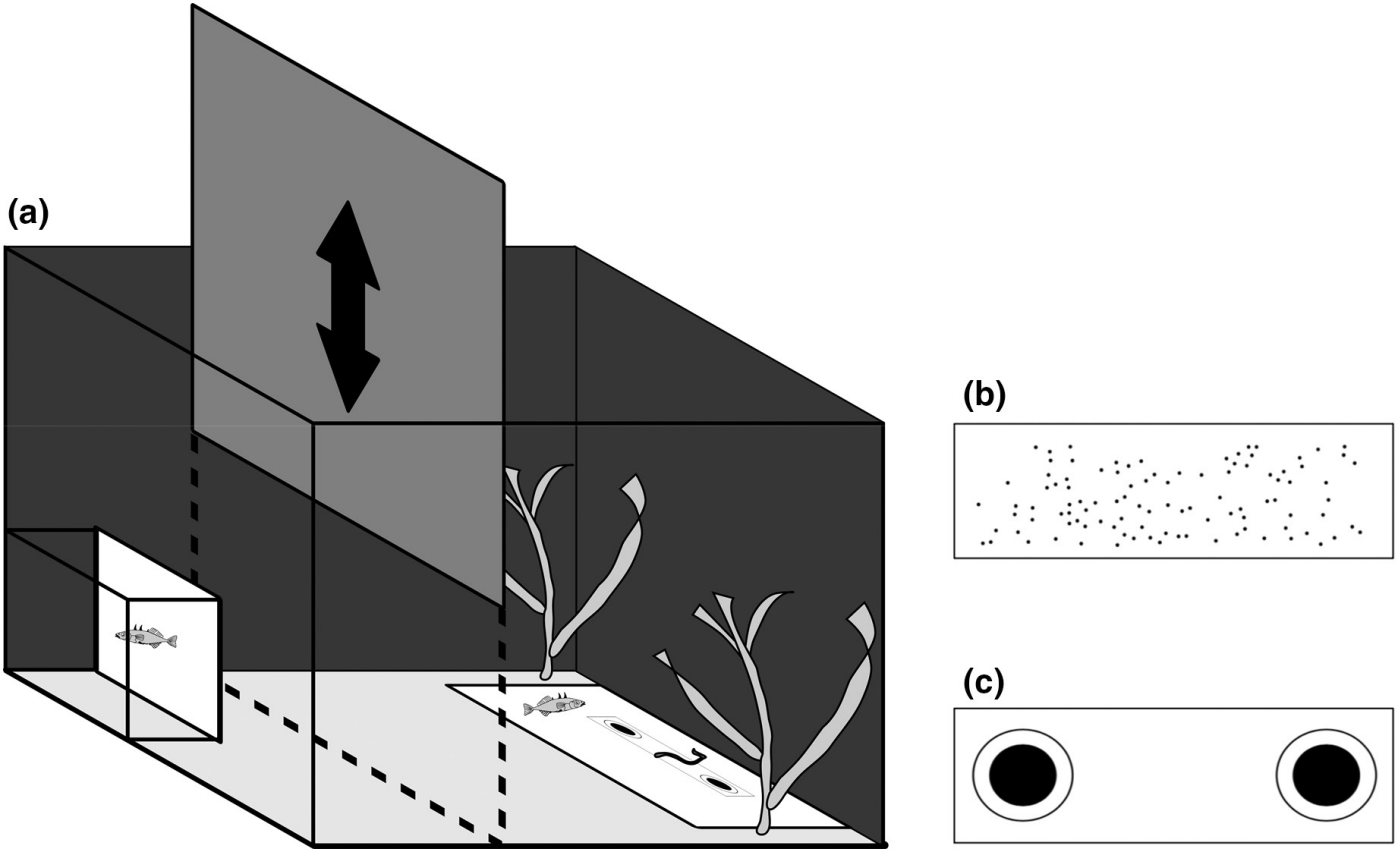
Schematic drawing of the experimental set‐up. (a) Fish were initially placed in the home compartment (left side in the diagram), partitioned from the test compartment (right side in the diagram) by an opaque divider operated by a pulley system. In the baseline trial, a bloodworm was placed on an empty feeding plate by the wall furthest from the home compartment. In the response trial, an artificial prey patterned with either (b) a control pattern or (c) a pair of eyespots was placed on the feeding plate with food centered on it. The companion fish was placed in a plastic container to prevent it from participating in the experiment. The divider was opened by a pulley system, allowing the focal fish to enter the test compartment to find and eat the bloodworm

The experiment was carried out in 28 identical 30‐L glass aquaria (L × W × H: 40 × 25 × 30 cm, Figure 1), with gravel on the bottom, plastic plants for hiding opportunities, and black plastic covering the external sides of the tanks (to avoid visual distraction and to better control the light conditions inside each aquarium). Each aquarium could temporarily be partitioned width‐wise with a removable opaque divider (25 × 30 cm), splitting the tank into a *home compartment* (approximately one third of the tank) and a *test compartment* (approximately two third of the tank, Figure 1). A white plastic plate (14 × 10 cm), henceforth called the *feeding plate*, was placed on the bottom of the test compartment by the aquarium wall furthest from the home compartment (Figure 1). The size and setup of these experimental aquaria closely match previous experiments assessing responses to eyespot and other visual stimuli in sticklebacks (Kjernsmo & Merilaita, 2013, 2017; Kjernsmo et al., 2016, 2019), facilitating a comparison of our results with previous findings. Three mirrors were attached along the tank's far side to provide a dorsal view over the test compartment, allowing an experimenter (E.J.) to visually monitor the experiment while minimizing the risk of disturbing the fish. Additionally, a digital camera (GoPro HERO 8 Black; GoPro, Inc.) was attached to the aquarium, providing recordings from above.

Our aim was to examine whether the visual environment influences behavioral responses in sticklebacks. However, examining specific, ecologically relevant visual environments is challenging. Along the Baltic Sea coast, and in other aquatic environments, underwater light intensity varies depending on water depth, season, and biotic factors (e.g., phytoplankton abundance, Lindström, 2000). Yet, despite this variation, light levels consistently change with the time of day. Regardless of the time of year, the underwater light intensity from coastal sites around the Baltic Sea is highest around solar noon, with light intensity being roughly 40–50% lower than the daily high in the 2 h following sunrise and before sunset (Lindström, 2000). Therefore, to test if light influences the function of eyespots, we performed the experiment in two different light environment treatments – low vs. high light – that broadly mimic daily changes in light levels. Adjustable light was provided by placing dimmable RGB LED light strips (Jula AB, Skara, Sweden) above each tank. Black plastic sheets were erected around the experimental tanks to minimize light leakage among the experimental tanks. Light brightness was measured inside each tank prior to each experimental trial by photographing a white standard placed on the feeding plate and quantifying brightness (measured in arbitrary units) using ImageJ (bundled with Java 1.8,0_172). The difference in brightness between the treatments was calculated by using the average brightness for all trials within each treatment. The difference in brightness between the two treatments was 106.7 units, and the low‐/high‐light ratio was ~0.43, i.e., the low‐light treatment's brightness was 43% of the high‐light treatment. This difference in brightness between the treatments can be interpreted as roughly equivalent to the difference in light intensity at mid‐day compared to either just after sunrise or just before sunset.

**FIGURE 2:**
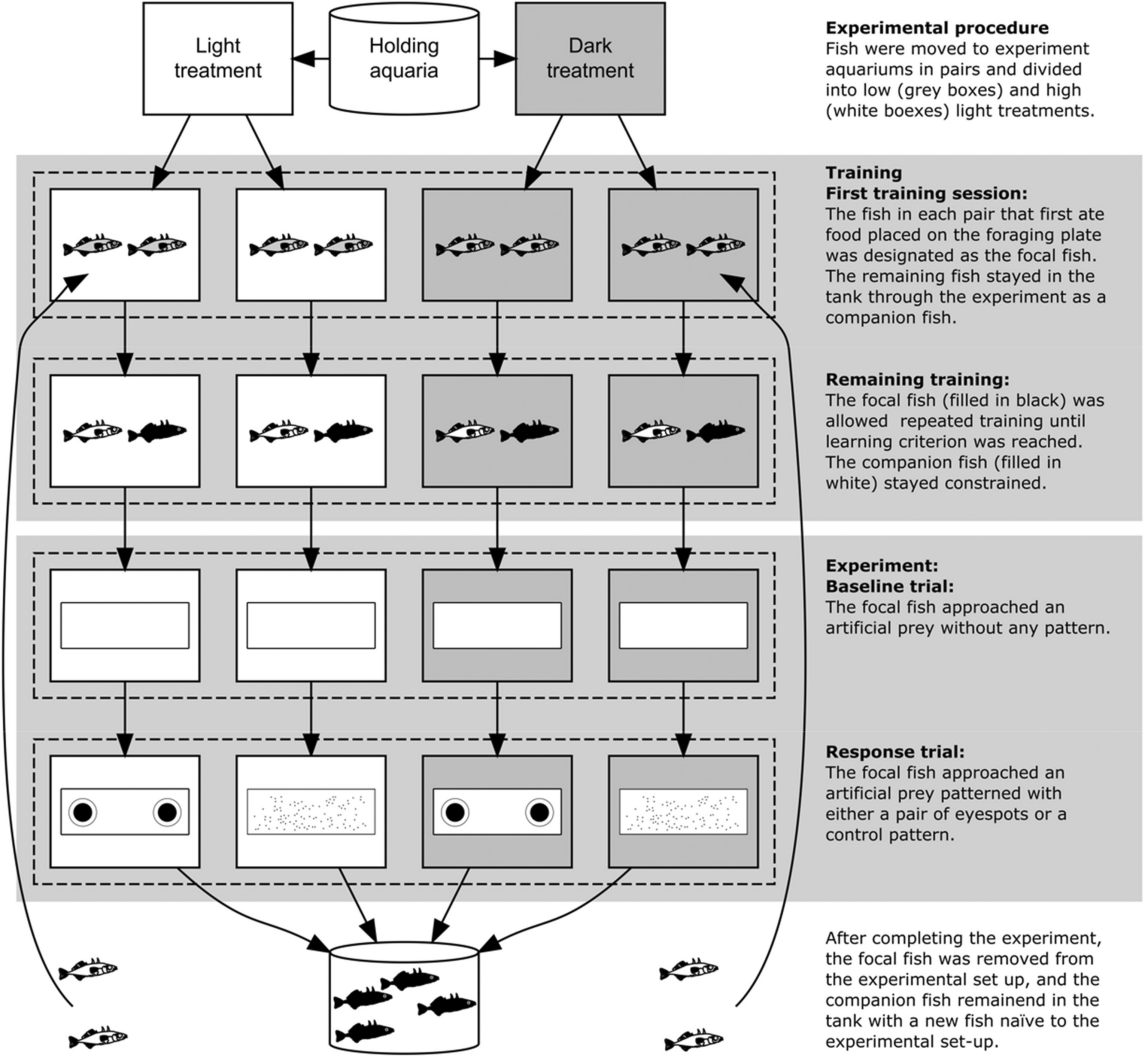
Flow diagram illustrating the training and experimental procedure. Sticklebacks were taken from their holding aquaria and divided into treatment groups (see Experimental procedure); here represented as gray boxes for the low light treatment and white boxes for high light treatment. Fish were transferred in pairs and allowed 3 days of acclimatization. The experiment then consisted of a training and experimental component for the sticklebacks. During the training component (see training), fish underwent a first training session where a focal fish (black fish) and a companion fish (white fish) were identified. The training was repeated during the remaining training sessions. When the learning criterion was reached, fish entered the experimental component (see Experiment). During a baseline trial, the focal fish was presented with food on the feeding plate and we recorded the fish's behaviors. During the response trial, fish were assigned a prey pattern stimulus (eyespot or control) and their behaviors were recorded. After the experiment, the focal fish were moved to a holding tank and the companion was paired with a new fish (indicated by dotted arrows) and the training and experimental component was restarted. Companion fish were used for maximum two times before being removed if not becoming the focal fish itself

We transferred fish from two holding aquaria (with 150 and 250 individuals in 450‐L and 600‐L aquaria with similar conditions as before) to the 30‐L experiment aquaria where they were acclimatized for 3 days before any experimental procedures (Figure 2). Our preliminary observations revealed that pairs of sticklebacks showed fewer behavioral signs of stress (e.g., low activity or frantic swimming behavior, reduced appetite) than sticklebacks held in isolation (personal observation). Therefore, fish were held in the experiment aquaria in pairs throughout the experiment to avoid the negative effects of habitual stress on feeding activity (e.g., Hovel et al., 2015). One fish in each pair, visually identified by phenotypic characteristics, was designated as the “*focal fish*” for the experiment. The second fish remained in the aquarium and was designated as the “*companion fish*” (see below and Figure 2). During the 3‐day acclimation period, fish were fed with bloodworms twice a day without any additional handling. After 3 days of acclimatization, all subsequent food was placed on the feeding plate. When the food had been obtained from the feeding plate within 15 min in two subsequent feedings, it was assumed that at least one of the fish was acclimatized enough to perform the experiment and could proceed to the training described below. If food remained on the feeding plate for more than 15 min for eight feedings (i.e., 4 days), both fish were removed from the study (this was the case for a total of 26 of 119 fish).

### 2.3 Training

All fish received training sessions in order to associate the test compartment with feeding opportunities and the feeding plate with food (summarized in Figure 2). An opaque divider was added to the experimental aquarium, with both fish located in the home compartment, separated from the test compartment. Food (a bloodworm) was placed on the feeding plate with forceps (note that the feeding plate was white [i.e., no pattern was present] during the training session). After a habituation period of at least 10 min, the opaque divider was lifted via a pulley system, allowing the fish to enter the test compartment and find the food on the feeding plate. In the first training session, both fish, distinguished by phenotypic characters, were allowed to enter the test compartment. The fish that first ate the food on the feeding plate was designated as the focal fish used in the experiment. The remaining fish, now the companion fish, was isolated during the following training sessions and later during experiment trials (and was fed after the experimental trial ended). The next training session was performed in the afternoon on the same day, and the experimental tank was again partitioned with the opaque divider with both fish in the home compartment. In the home compartment, the companion fish was placed in a plastic container (10 × 6.5 × 10 cm) with an opaque side that faced toward the test compartment. Thus, the companion fish was both physically and visually isolated from the ongoing experiment and was prevented from entering the test compartment in search of food, and thereby disrupting the trial of the focal fish. The session started when the divider was opened, allowing the focal fish to enter the test compartment and search for food, and ended when the focal fish ate the food or 15 min had passed. The companion fish was then released and both fish had access to the whole experiment tank until the next session. This procedure was repeated for a minimum of four sessions in total. When the focal fish completed a session within 1 min, on two consecutive sessions in a day, they were considered to have reached the learning criterion of the training and entered experimental trials the following day.

### 2.4 Experimental procedure

We observed stickleback behavior during the experiment when approaching and obtaining food (a bloodworm) on an artificial prey item with either eyespots or a control pretty pattern. The artificial prey consisted of a white rectangular piece of water‐proof paper (3.4 × 1.6 cm), with eyespots or the control pattern printed in black (Figures 1 and 2), which was placed on the feeding plate in both treatment groups. The eyespot prey pattern consisted of two symmetrical concentric rings (eyespot size = 6 mm in diameter, pupil size = 4 mm in diameter), oriented so that it appeared like a pair of eyes facing toward the fish. We used a pair of eyespots as paired stimuli can be more effective at deterring attacks (Mukherjee & Kodandaramaiah, 2015; but see Kjernsmo & Merilaita, 2017). To account for the novelty of the artificial prey itself, the control prey pattern consisted of a contrasting speckled pattern of 100 dots (0.06 mm in diameter, Figure 1). Thus, the total amount of black area and the black‐to‐white ratio on the artificial prey were the same in both the eyespot and control prey pattern treatments (sensu Kjernsmo & Merilaita, 2013).

The experiment itself consisted of two trials – a baseline trial (i.e., an internal control) and a response trial – that were broadly performed in the same way (Figure 2). As in the procedure in the previous training phase, the focal fish was placed behind an opaque divider in the home compartment, while the companion fish was placed in a plastic container in the home compartment. In the first trial, henceforth called the *baseline trial*, food (a bloodworm) was placed on the feeding plate without any artificial prey item underneath (i.e., the feeding plate was white, Figure 2). After a habituation period of at least 5 min, the opaque divider was lifted via a pulley system, allowing the focal fish to enter the test compartment, thereby initiating the trial. The trial ended when fish consumed the food on the feeding plate. The companion fish was released, and both fish had access to the whole experimental tank until the next trial, performed around 4–5 h later to ensure the fish regained hunger motivation. For the second trial, henceforth called the *response trial*, the focal fish was again placed in the home compartment behind the opaque divider while the companion fish was placed in a plastic container in the home compartment. This time, an artificial prey patterned with either a pair of eyespots or the control was placed on the feeding plate with food centered on it (*n* = 46 and 47 fish for the eyespot and control treatments, respectively, Figure 2). The trial was initiated when the focal fish entered the test compartment and ended when it attacked the prey item. By comparing differences in behavior each fish had between the baseline trial and the response trial, we could assess each fish behavioral response toward its assigned artificial prey pattern (eyespot/control). Thereby, we accounted for possible inter‐individual variation that was not related to the artificial prey pattern (e.g., personality, swimming speed, etc.).

To assess if behavioral responses toward eyespots are influenced by the light levels, the experiment was performed in two different light environment treatments. Approximately half of the fish performed training and experiment in the low‐light treatment (*n* = 47: *n* = 23 in the eyespot prey pattern treatment and *n* = 24 in the control prey pattern treatment), while the remaining fish were trained and performed the experiment in the high‐light treatment (*n* = 46: *n* = 23 in the eyespot prey pattern treatment and *n* = 23 in the control prey pattern treatment).

After completing the experiment trial, the focal fish was replaced with a new fish naïve to the experimental set‐up (Figure 2). The companion fish stayed in the experimental tank with the new fish, acting as either a focal fish or companion fish depending on the outcome of the first training session, but only for a maximum of three experimental trials (Figure 2). This resulted in an assemblage of pairs of fish with different experiences in the experimental set‐up (i.e., some fish had previous experience as companion fish while others did not), which was accounted for in subsequent statistical analyses (see below). If neither fish left the home compartment within 15 min in an experimental trial, they were both removed from the study. A total of 93 fish successfully completed the experimental trials.

### 2.5 Behavioral observations

Each trial was observed in real time by E.J. through mirrors attached above the tank. Feeding latency, defined as the time from when the focal fish entered the test compartment to when it consumed the bloodworm, was recorded in real time using a stopwatch. It was challenging to quantify the number of stops a fish made during the trial, defined as the number of times a fish stayed motionless except for moving its' pectoral fins, in real time, particularly in the low‐light treatment. Therefore, the number of stops fish made was determined from video recordings of the trials. Due to visual constraints in the low‐light treatment, we were unable to obtain data from some of the trials. However, we could unambiguously quantify the number of stops a fish made in 89 of the 93 trials and used data obtained from these video recordings in our analyses.

### 2.6 Statistical analysis

All statistical analyses for this experiment were conducted in Rstudio version 1.2.5001. To account for inter‐individual variance in behavioral responses among fish, we calculated the difference in the feeding latency and number of stops between the response trial and the baseline trial and used these values as the dependent variables in our models. We conducted linear models to test if behavioral responses (difference in feeding latency and number of stops) of sticklebacks were influenced by prey pattern (eyespot/control), light environment treatment (low/high light), if fish acted as a companion fish before its experiment trial (yes/no), and by the interactions among these independent variables.

First, we used the *lm* function in the R package *stats* to fit our models, including all the independent variables we expected to influence the dependent variables. We then performed an automatic stepwise refinement using the *stepAIC* function in the R package *MASS*, which uses a bi‐directional AIC‐based heuristic (it removes and adds predictors based on values of the Akaike information criterion) in order to find the subset of variables that best explain the variation in the dependent variable. Finally, we fitted the model with the lowest prediction error from the stepwise refinement using the *lm* function.

Two outlier data points in our data set were identified and excluded from further analysis after visual inspection of the model residuals, reducing the sample size to 91 for feeding latency and 87 for stops. These data points deviated markedly from other observations, one in each direction, but their exclusion did not change the overall statistical pattern. The outcome of a statistical analysis with a full data set is provided in Table S1.

## 3 RESULT

**FIGURE 3:**
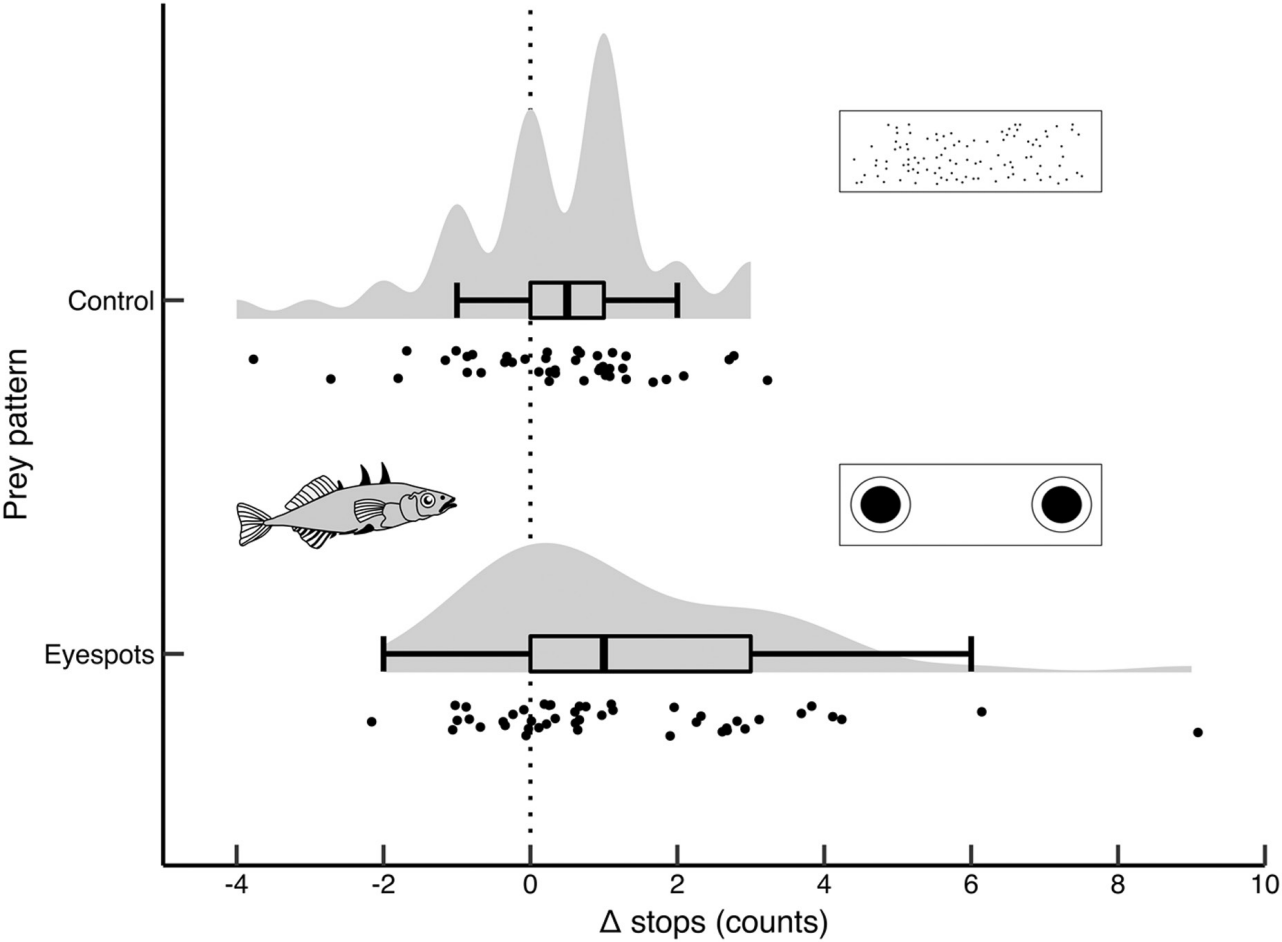
Raincloud plot showing the effect of prey pattern on the difference in the number of stops before feeding in the response – Baseline trial. The dotted vertical line through zero indicates no difference in behaviors in the response and baseline trials, while positive and negative values indicated that fish stopped more or less, respectively, during the response trials. Distributions of responses are presented as unmirrored violin plots, combined with boxplots showing medians (solid lines), with lower and upper hinges corresponding to first and third quartiles (IQR), and whiskers to the largest and smallest values within 1.5 (IQR). Vertical jitter below the boxplots represents individual data points

The number of stops fish made when approaching the food was significantly influenced by prey pattern (Table 1). Specifically, sticklebacks stopped more than four times as often (on average and compared to the baseline trial) when approaching prey with eyespots compared to prey with control patterns (Table 1, Figure 3). There was a statistical trend suggesting that the number of stops fish made was influenced by an interaction between prey pattern and experience as a companion fish (Table 1). Post hoc examination of this interaction suggested that fish that had not been companion fish (i.e., less experienced fish) made more stops when approaching eyespots (compared to the baseline trial), while stop number was not influenced by prey pattern in fish that had been companions (i.e., more experienced fish) (see Figure S1 for a plot of this interaction effect). The number of stops made during the trials was not influenced by light environment treatment or by an interaction between light environment treatment and prey pattern (Table 1).

**TABLE 1:** Effects of prey pattern (eyespot/control), light (low/high), and experience as a companion fish (yes/no) on sticklebacks behavioral responses (feeding latency and stops)

Predictors	Feeding latency				Stops			
	Estimate	CI	*t*	*p*	Estimate	CI	*t*	*p*
Intercept	14.20	0.44–27.96	2.05	.04	0.02	−0.88–0.92	0.04	.97
Prey pattern	4.94	−15.89–25.77	0.47	.64	1.82	0.52–3.11	2.79	.01
Light	−16.53	−34.71–1.64	−1.81	.07	0.15	−0.98–1.28	0.27	.79
Companion fish	2.53	−18.01–23.07	0.24	.81	0.73	−0.52–1.97	1.16	.25
Prey pattern * Light	4.20	−22.11–30.51	0.32	.75	−0.67	−2.28–0.94	−0.82	.41
Prey pattern * Companion fish	−21.06	−50.52–8.41	−1.42	.16	−1.75	−3.53–0.02	−1.97	.05
*N*	91				87			
*r* ^2^/*r* ^2^_adjusted_	0.08/0.02				0.11/0.06			

*Note*: The linear models represent fitted models with the lowest prediction error from the stepwise refinement. The estimate, confidence intervals (CI), *t*‐value, and *p*‐value, sample size and *r* ^2^/*r* ^2^_adjusted_ values are presented for each model.

Feeding latency increased when fish made more stops as they approached the food (*t* = 5.23, *p* < .001). However, feeding latency was not influenced by prey pattern, experience as a companion fish, or by the interactions between prey pattern and light environment treatment, and prey pattern and experience as a companion fish (Table 1). However, there was a statistical trend suggesting that feeding latency increased (compared to the baseline trial) when fish were in the low‐light treatment (Table 1).

## 4 DISCUSSION

In this study, we show that sticklebacks made more stops when approaching prey patterned with a pair of eyespots than when approaching a prey patterned with a control stimulus. This behavioral response appears to be innate rather than learned, as our experimental design focused solely on the fish's first (and only) encounter with the eyespot stimuli. Critically, behavioral responses to eyespots could not be explained by differences in the black‐to‐white ratio of the eyespot and control stimuli, as we experimentally equalized this ratio between prey pattern treatments. However, contrary to our prediction, we did not observe an increase in feeding latency in the presence of eyespots. Thus, while sticklebacks may stop more often as they approach eyespot stimuli, the total amount of time required to eat the prey item does not vary between eyespot and control stimuli. Moreover, contrary to our prediction, behavioral responses to eyespots were consistent in both the low‐ and high‐light treatments. However, it is currently unclear whether light levels do not influence behavioral responses or if the light levels used in our experiment were insufficient to warrant a differential response. Overall, the result provides evidence that a pair of eyespots can modulate the behavior of an aquatic predator, although these effects vary depending on the specific behavior being considered.

When sticklebacks approached an artificial prey with a pair of eyespots, they made more stops than sticklebacks approaching prey with a control pattern. This result is consistent with expectations from the intimidation hypothesis, and mirrors the hesitation observed in some terrestrial species when attacking stimuli with eyespots (e.g., great tits, *Parus Major*, and attacking peacock butterflies, *Junonia almana*, Kodandaramaiah et al., 2009). Importantly, the eyespots used in our experiment were designed specifically to be larger than the fish's own eye (Kjernsmo & Merilaita, 2013). Thus, if stickleback perceived eyespots as belonging to a natural predator – like a perch, for example, – then this may explain their apparent caution in approaching the eyespot stimuli. When confronted with a potential fish predator, sticklebacks slowly approach the predator with frequent stops and starts (Östlund‐Nilsson et al., 2006). This response is described as “predator inspection” behavior, where the approach allows fish to continually update the information on how much of a threat the potential predator poses and therefore whether it is safe to continue with other activities such as feeding (Östlund‐Nilsson et al., 2006). Similarly, in experimentally controlled encounters, sticklebacks respond to a nearby and approaching model predator by increasing the amount of time spent motionless (Näslund et al., 2016). Together, this suggests that the more frequent stops induced by eyespots in our experiment may reflect an increase in anti‐predator behaviors in sticklebacks.

Contrary to expectation, eyespots did not influence the feeding latency in our experiment. This result was surprising as there was a positive relationship between feeding latency and the number of stops sticklebacks performed while approaching the prey, suggesting that these two behavioral should respond similarly to the eyespot stimuli. One possibility is that the experimental tanks used in this study were too small to uncover an effect on feeding latency. If so, then future studies should consider using larger experimental tanks that more closely mirror ecological conditions experienced by sticklebacks. However, the lack of an effect on feeding latency is consistent with the result of a similar study by Kjernsmo & Merilaita (2013), who also found that feeding latency did not differ when sticklebacks were exposed to either a single eyespot or a control pattern (but see Kjernsmo & Merilaita, 2017 for an example of eyespots increasing feeding latency in sticklebacks). The lack of an effect on feeding latency is consistent with the deflection hypothesis, where eyespots function to draw attacks toward non‐vital body parts (Kjernsmo & Merilaita, 2013). This explanation, however, seems less relevant in our study since we examined large eyespots that should be less likely to draw attacks from sticklebacks. Instead, small eyespots, which are often located on marginal body parts, are more likely to play a functional role in deflecting predator strikes (Vallin et al., 2011). Alternatively, our experimental setup may not have provided adequate stimuli, such as movement, to trigger responses in feeding latency to eyespots. For example, continuously moving predator stimuli induced stronger anti‐predator related behavioral responses in sticklebacks (Näslund et al., 2016). Finally, behavioral responses to eyespots are more extreme in sticklebacks that were previously exposed to visual and olfactory cues of predation (Kjernsmo & Merilaita, 2017), suggesting that the predator naïve fish used in our experiment may have had muted reactions to eyespot stimuli. Investigating how moving eyespot stimuli and previous experience with predators influence the efficacy of eyespots in deterring attacks would help to clarify the function of eyespots in aquatic animals, while also being relevant for terrestrial study systems where the anti‐predator function(s) of eyespots remains unclear (e.g., Lyytinen et al., 2003).

The light environment in our experimental treatments did not influence how sticklebacks responded to eyespots. A possible explanation for this could be that aspects other than just light levels determine how an eyespot functions. For example, in a study by Olofsson et al. (2010), the woodland butterfly (*Lopinga achine*) were effective in deflecting attacks from blue tits (*Cyanistes caeruleus*) only in low‐light conditions with prominent UV levels. This effect is explained due to a shift of attention in the perception of the prey, where the increased salience of the UV‐reflective white pupil emphasizes the eyespot and fades the rest of the butterfly, thereby strengthening its deflective effect (Olofsson et al., 2010). Therefore, it is possible that other attributes, such as UV light, may operate similarly in water environments (e.g., Rick & Bakker, 2010). Additionally, it is possible that the light level in did not differ sufficiently between the light environment treatment to detect an effect. However, a statistical trend suggested that fish being slower to feed in the low‐light treatment implies that the difference in light levels between the treatments was at least was apparent for the fish. Nevertheless, it is possible that greater light modifications could lead to other outcomes. We propose that future studies investigating sticklebacks' response to eyespots should consider more variation in light conditions and potentially different attributes of their prey pattern.

To conclude, our results provide some support that eyespots may offer anti‐predator benefits in an aquatic environment. There is emerging evidence that eyespots function to reduce predation risk by both intimidating and deflecting predators. Specifically, the results from this study provide evidence that paired eyespots can manipulate behaviors of sticklebacks, causing them to stop more often when approaching a prey. If increases in stopping represent an anti‐predator behavior in sticklebacks, as has been argued (Östlund‐Nilsson et al., 2006), then eyespots may function to intimidate potential predators. An intimidation function of eyespots in aquatic environments could help to explain cases where eyespots are located centrally on the bodies of aquatic animals (Helfman & Burgess, 2014; Hemingson et al., 2021; Motta, 2012). Yet, eyespots – particularly, when small and located on the marginal body parts – also appear to play a role in deflecting attacks in fishes (e.g., Winemiller, 1990; Kjernsmo & Merilaita, 2013). Eyespots may play a different functional role in reducing predation risk depending on the species and predatory–prey dyad being considered. However, our results clearly highlight the potential importance of including several behaviors when observing anti‐predatory responses of eyespots, both in aquatic and terrestrial environments.

## AUTHOR CONTRIBUTIONS

**Evelina Juntorp:** Formal analysis (equal); investigation (lead); methodology (lead); visualization (equal); writing – original draft (equal). **Madicken Åkerman:** Conceptualization (equal); visualization (equal); writing – original draft (equal). **J. L. Fitzpatrick:** Conceptualization (equal); funding acquisition (lead); project administration (lead); supervision (lead); writing – review and editing (lead).

## CONFLICT OF INTEREST

None declared.

## Supporting information


Appendix S1

Appendix S2

Appendix S3

## Data Availability

The raw data and R script used to analyze to generate the results here are provided in the Supplementary Material Appendices S1 and S2. The raw data file and R script are available on Dryad (https://doi.org/10.5061/dryad.9p8cz8wk5).
