# Supplementary material for: Are behavioral responses to eyespots in sticklebacks influenced by the visual environment? An experimental examination
Source: Ecol Evol. 2022 Jul 5;12(7):e9089. doi: 10.1002/ece3.9089 (PMC9256514; doi:10.1002/ece3.9089)

Supplemental Material

Are behavioural responses to eyespots in sticklebacks influenced by the visual environment? An experimental examination

Evelina Juntorp^1†^, Madicken Åkerman^1†^, John L. Fitzpatrick^1^*

^1^Department of Zoology: Ethology, Stockholm University, Svante Arrhenius väg 18b, 106 91 Stockholm, Sweden.

†These authors contributed equally.

*Corresponding Author: email [john.fitzpatrick@zoologi.su.se](mailto:madicken.akerman@zoologi.su.se)

**Supplementary Figure S1.**


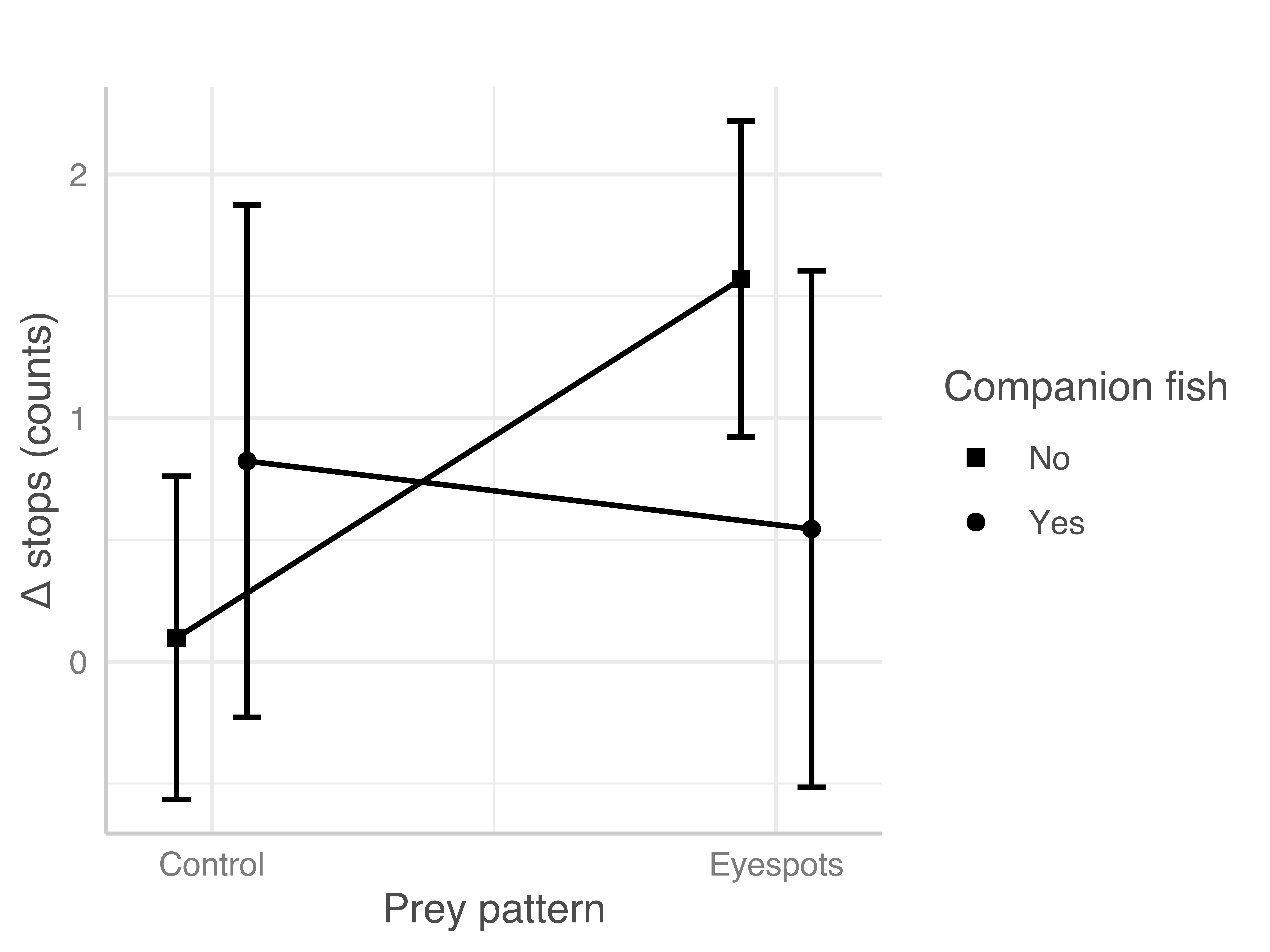


**Supplementary Figure S2. Interaction plot between prey pattern (control vs eyespots) and companion fish (no vs yes).** A statistical trend suggests that fish with no experience as a companion made more stops approaching a prey with eyespots. Fish with experience as a companion showed no difference in number of stops.

**Supplementary Table S1.**

Effects of prey pattern (eyespot/control), light (low/high) and experience as a companion fish (yes/no) on sticklebacks behavioural responses (feeding latency and stops) with a dataset including the two outliers removed in the main analysis. The linear models represent fitted models with the lowest prediction error from the stepwise refinement.


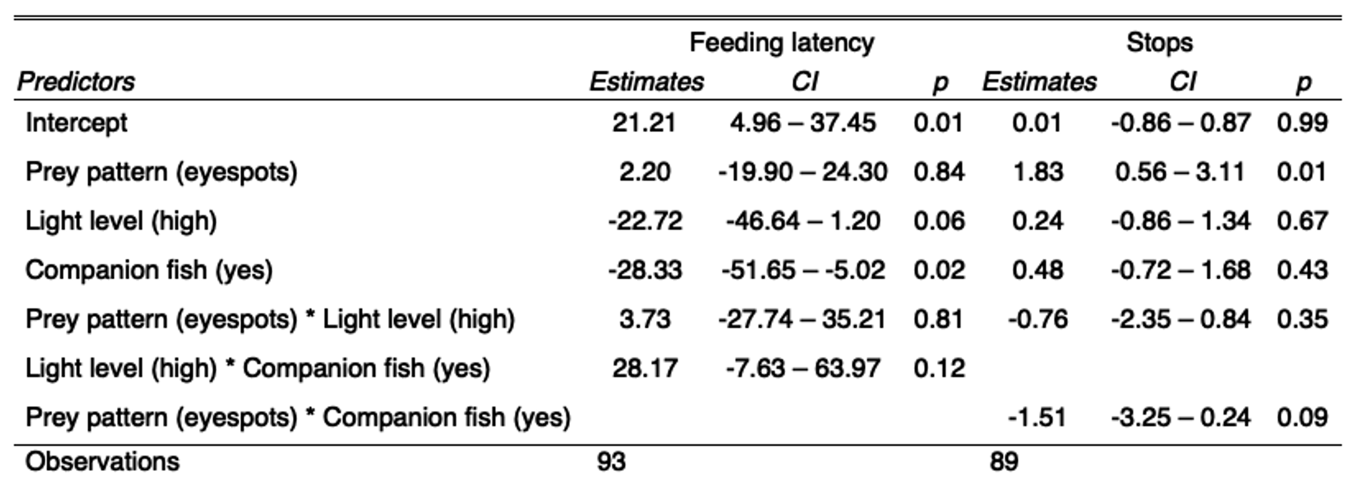

Supplement: Supplementary file 3 — Appendix S3 [file ECE3-12-e9089-s002.docx]
